# Bite by byte: can fitness wearables help bariatric patients lose more weight after surgery?

**DOI:** 10.1007/s00464-023-10157-z

**Published:** 2023-06-07

**Authors:** Estella Y. Huang, Daniel Chung, Hannah M. Hollandsworth, Nicole H. Goldhaber, Lorijane Robles, Maria Horgan, Bryan J. Sandler, Garth R. Jacobsen, Ryan C. Broderick, Eduardo Grunvald, Santiago Horgan

**Affiliations:** 1 Division of Minimally Invasive Surgery, Department of Surgery, Center for the Future of Surgery, University of California, 9500 Gilman Drive, MET Building 845, La Jolla, San Diego, CA 92093-0740 USA; 2 Division of General Internal Medicine, UCSD Bariatric and Metabolic Institute, University of California San Diego, La Jolla, San Diego, CA USA

**Keywords:** Fitness wearable, Bariatric surgery, Lifestyle modification

## Abstract

**Background:**

Multidisciplinary approaches to weight loss have been shown to improve outcomes in bariatric patients. Few studies have been performed assessing the utility and compliance of fitness tracking devices after bariatric surgery. We aim to determine whether use of an activity tracking device assists bariatric patients in improving postoperative weight loss behaviors.

**Methods:**

A fitness wearable was offered to patients undergoing bariatric surgery from 2019 to 2022. A telephone survey was conducted to elucidate the impact of the device on the patient’s postoperative weight loss efforts 6 to 12 months after surgery. Weight loss outcomes of sleeve gastrectomy (SG) patients receiving the fitness wearable (FW) were compared to those of a group of SG patients who did not receive one (non-FW).

**Results:**

Thirty-seven patients were given a fitness wearable, 20 of whom responded to our telephone survey. Five patients reported not using the device and were excluded. 88.2% reported that using the device had a positive impact on their overall lifestyle. Patients felt that using the fitness wearable to keeping track of their progress helped them both to achieve short-term fitness goals and sustain them in the long run. From the patients that utilized the device, 44.4% of those that discontinued felt like it helped them build a routine that they maintained even after they were no longer using it. Demographic data between FW and non-FW groups (age, sex, CCI, initial BMI, and surgery BMI) did not differ significantly. The FW group trended towards greater %EWL at 1 year post-operation (65.2% versus 52.4%, *p* = 0.066) and had significantly greater %TWL at 1 year post-operation (30.3% versus 22.3%, *p* = 0.02).

**Conclusion:**

The use of an activity tracking device enhances a patient’s post-bariatric surgery experience, serving to keep patients informed and motivated, and leading to improved activity that may translate to better weight loss outcomes.

The modern age of digital technology has allowed for huge improvements in personal health management. It has allowed patients to have access to a wealth of digestible information about their daily lives, improving awareness and accountability. One example of this is the use of smart fitness wearable devices, which have biometrical sensors that can continuously generate real-time health information for the user.

One population that can potentially benefit from this is bariatric patients. Weight regain after bariatric surgery has been reported to be as high as 35%, though its definition is inconsistent across different studies [1]. While the causes are multifactorial, behavioral factors likely contribute [2, 3]. This can include maladaptive eating, drug and alcohol abuse, and lack of adequate physical activity.

On the other hand, self-monitoring behaviors have been found to be protective against weight regain after bariatric surgery [4]. A fitness wearable can aid with behavior modulation, which can translate to long-term weight maintenance. We aim to study the use of an activity tracking device in improving post-bariatric surgery weight loss behaviors.

## Methods

A fitness wearable was offered to patients undergoing bariatric surgery between 2019 and 2022. The participants for the study were chosen at random, but who expressed interest and commitment during study enrollment. Demographic data included age, sex, Charlson Comorbidity Index (CCI), initial BMI (at time of entry to the bariatric program) and BMI on day of surgery. Device data included frequency of use, and use of features such as diet log, weight log, and sleep tracking. Six to 12 months after surgery, a telephone survey was conducted to elucidate the impact of the device on the patient’s postoperative weight loss efforts. Transcripts were blinded and coded by three separate reviewers to find common themes and conclusions.

Sleeve gastrectomy (SG) patients receiving the fitness wearable (FW) were compared to a group of SG patients who did not receive one (non-FW). Non-FW patients were chosen randomly from a database of sleeve gastrectomy patients (excluding those who were offered a fitness wearable). They were called to confirm that they did not use any fitness wearables during the study period. If they used a fitness wearable at any point in time during the study period, they were excluded. Percent excess weight loss (EWL) and percent total weight loss (TWL) outcomes were compared between the groups at 2 weeks, 6 weeks, 3 months, 6 months, 9 months, and 1 year postoperatively. Percent EWL was calculated using the following formula: (initial visit weight–current weight)/excess weight, with excess weight being defined as initial visit weight–ideal weight (corresponding to BMI 25). Percent TWL was calculated using the following formula: (initial visit weight–current weight)/initial visit weight.

Student’s *t*-test was used for continuous variables with parametric distribution, Wilcoxon rank-sum test was used for continuous variables with nonparametric distribution, and Fisher’s exact test was use for categorical variables. All analyses were performed in R (Version 4.1.3, Vienna, Austria). Statistical tests were two-sided and a p-value of ≤ 0.05 was considered significant.

## Results

A total of 108 sleeve gastrectomies were performed over the course of the study period. Thirty-seven patients were given a fitness wearable, 20 of whom responded to our telephone survey. Five patients reported not using the device and were excluded. Five patients (31.3%) started using the device prior to surgery, the rest started using the device after surgery, mostly within the first postoperative month. On average, patients reported using it for 6.4 months (range 0.75 to 18 months) for 6.4 days weekly (range 4–7 days) with 9 (56.3%) reporting that they still currently use a fitness wearable, either the one provided for them or a different one.

Patients reported that the device motivated them to build healthy habits by keeping track of their progress in exercise in addition to other areas such as sleep and food and water intake. Seventy-five percent of patients reported that having daily reminders to exercise, eat healthy, and sleep more helped them carry out these actions more consistently. Among the patients surveyed, 88.2% reported that using the device had a positive impact on their overall lifestyle with 88.2% reporting better exercise habits and 58.8% reporting that it assisted with their weight loss. In addition to the reminders, many patients found the device helpful because it kept track of their exercise progress. Among users, 93.8% reported that they mainly used their device to set daily step goals and count them, with 75% reporting that keeping track of their exercise in this way was the most helpful part of using the device. All patients who used this device feature felt motivated to consistently meet their daily step quota with a majority feeling emboldened to surpass their daily step goals in ensuing exercise routines. Some patients felt that keeping track of their exercise progress in this objective manner helped them realize they could not only achieve their short-term fitness goals but also sustain them in the long run. From the patients that utilized the device, 44.4% of those that discontinued felt like it helped them build a routine that they maintained even after they were no longer using it.

All participants who used the device reported that it was user-friendly, with 88.2% reporting that they would want to continue using it. All patients said they would recommend it to other patients, with some even purchasing it for a family member or friend as a gift. Yet while many found it useful, the device certainly had its limitations. Of the 9 patients who stopped wearing it, 3 (33.3%) discontinued use of the wearable due to its limited charging capacity, while another 33.3% stopped wearing it due to band malfunction and discomfort. Two (22.2%) patients resorted to a different wearable, with both citing that the other device’s functionalities fit their lifestyle needs better.

### Subgroup analysis: comparison to non-FW

**Fig. 1:**
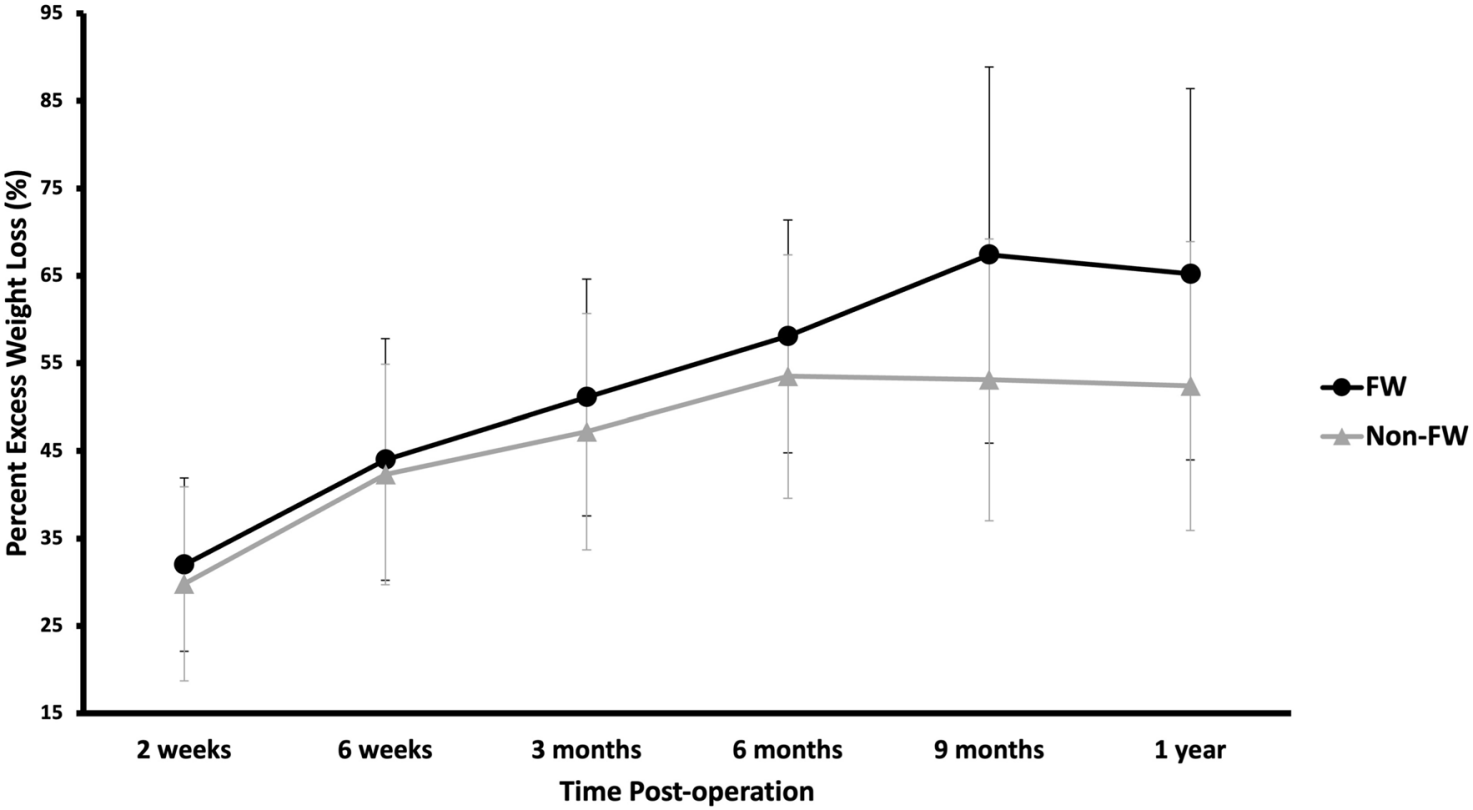
Postoperative % excess weight loss in FW versus non-FW

**Fig. 2:**
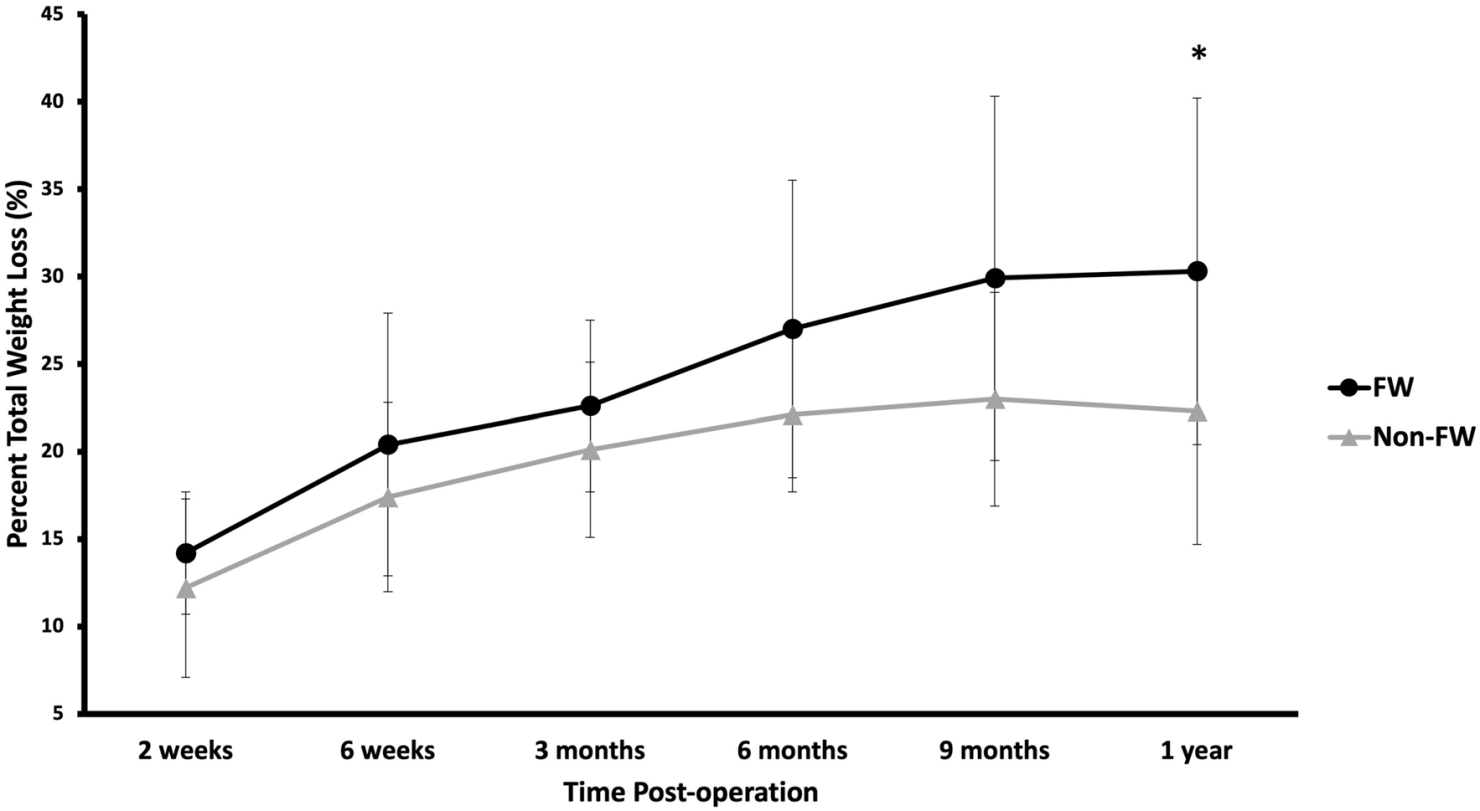
Postoperative % total weight loss in FW versus non-FW

The 15 FW patients were compared to a group of 24 non-FW patients. Demographic data (age, sex, CCI, initial BMI, and surgery BMI) did not differ significantly between groups (Table 1). The FW group trended towards more %EWL at 9 months (67.4% versus 53.1%, *p* = 0.09) and 1 year (65.2% versus 52.4%, *p* = 0.066) postoperatively (Table 2, Fig. 1). FW patients trended towards greater %TWL at 9 months (29.9% versus 23.0%, *p* = 0.08) and had significantly greater %TWL at 1 year (30.3% versus 22.3%, *p* = 0.02) (Table 3, Fig. 2).

**Table 1:** Demographics

	All (*n* = 39)	FW (*n* = 15)	Non-FW (*n* = 24)	*p*
Age, years (SD)	44.6 (14.0)	44.1 (11.3)	44.9 (15.7)	0.86
Female, n (%)	35 (89.7%)	13 (86.7%)	22 (91.7%)	0.63
CCI mean (SD)	1.0 (1.4)	1.1 (1.4)	0.9 (1.4)	0.55
0	20 (51.3%)	7 (46.7%)	13 (54.2%)	
1	11 (28.2%)	4 (26.7%)	7 (29.2%)	
2	2 (5.1%)	0	2 (8.3%)	
3 +	6 (15.4%)	4 (26.7%)	2 (8.3%)	
Initial BMI, kg/m^2^ (SD)	45.2 (7.0)	47.6 (8.1)	43.7 (5.8)	0.12
Surgery BMI, kg/m^2^ (SD)	40.1 (4.5)	41.3 (5.2)	39.4 (4.0)	0.23
Patients w/ Initial BMI ≥ 50, n (%)	10 (25.6%)	6 (40.0%)	4 (16.7%)	0.14

**Table 2:** Postoperative % excess weight loss in FW versus non-FW

Post-op	FW (*n* = 15)	*n*	Non-FW (*n* = 24)	*n*	*p*
2 weeks	32.0 (9.9)	13	29.8 (11.1)	18	0.56
6 weeks	44.0 (13.8)	14	42.3 (12.6)	16	0.72
3 months	51.1 (13.5)	13	47.2 (13.5)	20	0.42
6 months	58.1 (13.3)	13	53.5 (13.9)	18	0.36
9 months	67.4 (21.5)	10	53.1 (16.1)	15	0.09
1 year	65.2 (21.2)	14	52.4 (16.5)	23	0.066

**Table 3:** Postoperative % total weight loss in FW versus non-FW

Post-op	FW (*n* = 15)	*n*	Non-FW (*n* = 24)	*n*	*p*
2 weeks	14.2 (3.5)	13	12.2 (5.1)	22	0.18
6 weeks	20.4 (7.5)	14	17.4 (5.4)	20	0.22
3 months	22.6 (4.9)	13	20.1 (5.0)	22	0.16
6 months	27.0 (8.5)	13	22.1 (4.4)	19	0.07
9 months	29.9 (10.4)	10	23.0 (6.1)	16	0.08
1 year	30.3 (9.9)	14	22.3 (7.6)	24	0.02

## Discussion

Bariatric surgery patients using a fitness wearable found it to be helpful in motivating them to build a healthy routine in the immediate postoperative period and conducive to maintaining those healthy habits long-term. Fitness wearables provided users with a quantifiable method of tracking their activity and encouraged healthy habits through consistent reinforcement. When compared to patients who did not use one, device users trended towards greater postoperative weight loss.

There is conflicting evidence on whether the use of fitness wearables can translate to tangible benefits. A systematic review looking at over 20 studies reported that using a wearable device as a part of a multimodal program appeared to help with weight loss in middle-aged or older adults [5]. Most studies did not follow patients beyond 12 months with the majority of intervention times falling between 6 and 12 months. On the other hand, a randomized clinical trial investigating the addition of a wearable technology device to a behavioral intervention program found that it did not offer any advantage in weight loss [6]. However, this study was performed 10 years ago, and the device used (a wearable armband) does not reflect more contemporary devices that are worn on the wrist and have more advanced functionality. This study also looked primarily at younger patients (ages 18 to 36), which may demonstrate a lack of generalizability. Regardless, fitness wearables have become increasingly popular and have become a way that people can become more connected to both themselves and the world around them [7, 8].

While there is a plethora of studies on the use of fitness wearables in the general population, few focus specifically on its use in bariatric patients. In our cohort, patients using a fitness wearable trended towards better weight loss than those not using one. This may not have been a significant finding because the data reflects only the first postoperative year, during which the metabolic effects of bariatric surgery may predominate. Nevertheless, while behavioral factors may have less initial impact postoperatively, they are still pertinent in helping the patient to maintain durable weight loss [9, 10]. The benefits of exercise and nutrition counseling post-bariatric surgery have been studied extensively, with several studies reporting increased physical activity to be positively associated with better postoperative outcomes, including weight loss [11–14]. However, successful long-term behavior modification is contingent on the establishment of healthy habits, which may not be so easily achievable.

A study conducted to investigate how long it took for patients to form a health habit discovered that it took around 66 days. Habit formation time was dependent on type of habit and varied between individuals, taking up to 250 days in some cases. As expected, simple tasks became habits faster and more easily than more complex ones [15]. Adoption of a healthy lifestyle involves the development of a routine and the formation of several complex habits, which takes longer to accomplish. Features of a fitness wearable that can promote such healthy habit formation include an intervention system and connection to a community [16, 17]. It can provide users with personalized feedback based on their health data or send users proactive messages with expert advice. It also creates a platform for shared experiences and goals, helping to keep users more accountable. Additionally, because it provides a way for users to objectively measure and track their progress, it gives them a way to set tangible goals based on specific metrics [18].

Given that habits require time to form, the long-term sustainability of a fitness wearable needs to be addressed. Retention is low, with reports of the abandonment rate being up to 30% [19]. The integration of activity trackers into smart devices that have other functionalities (e.g., keeping the time and checking and sending messages) has helped to improve utilization over the years [16]. In our study population, around 60% of the patients were still actively using the device at the time of the interview, but the rest had stopped. Reasons for stopping varied, but a big factor could have been the lack of certain features that can improve useability and interest, like a high level of gamification or interactivity [20]. However, it is also important to note that around half of the patients who discontinued use continued healthy routines that they formed while still using the device. Almost all participants felt that the device had some positive impact on their habits, even those who reported that they stopped using it. This shows that the fitness wearable can have positive long-term effects despite cessation of use.

Most common reasons for not using any fitness wearables among the non-FW group included not believing that it would be helpful, not being aware that such a device existed, and inability to afford a device. While some patients in the FW group had prior exposure to fitness wearables, for many this was their first device. Almost all participants would recommend it to other patients, and some found it to be so helpful that they purchased one as a gift for friends or family. It is likely that fitness wearables will increase in popularity with the coming years as they become more accessible and versatile.

Limitations of this project include its single-center design and small sample size. The participants for the study were chosen at random, but also expressed interest and commitment during study enrollment. Although this approach may introduce a certain degree of bias in the selection of fitness wearable users and may not be representative of the entire bariatric population, we believe that this approach was best for randomization while also increasing the potential for good objective data from the devices in use. When identifying patients who would benefit from the device, we emphasize the importance of identifying individuals who express genuine interest and willingness to invest consistent effort in its usage from the start. As with any healthcare plan, the decision to utilize a fitness wearable should be tailored to each patient’s needs, and it is important to identify patients for whom this method would be a good fit. Sampling bias is also a consideration given that data for both groups were only able to be collected on patients who answered their phone. While several patients who were not interviewed did receive the device, they were not included in the analysis because use of the device was not able to be confirmed. Recall bias could also have influenced the data collected through phone interview. Finally, social desirability bias may have overestimated the benefits of the fitness wearable. This was a pilot study to test the feasibility of fitness wearable use in the bariatric population. Future directions for this project include expanding the number of patients and tracking patient progress for a longer period of time, as well as including activity data.

Fitness wearables use a combination of real-time feedback, community connection, and goal reinforcement to increase awareness and motivation. This should be harnessed in the bariatric population to help patients maintain healthy lifestyle habits post-surgery and can be a powerful tool in helping to maintain weight loss. With continuous improvements in technology and device design, the use of activity tracking devices will have longer-term retention and can increase accessibility to health services, and possibly even lower healthcare costs. Conversely, if fitness wearables do not demonstrate added benefit for weight loss and other health measures, excess cost, effort, and technology burden might be avoided.

## Conclusion

While larger, longer-term studies are warranted to further define the benefits of fitness wearables, this study underscores their potential in enhancing the post-bariatric surgery experience, serving to keep patients informed and motivated, and leading to improved activity that can possibly translate to better weight loss outcomes.
